# Strong attractive interaction between finite element models of twisted cellulose nanofibers by intermeshing of twists†

**DOI:** 10.1039/d3ra01784b

**Published:** 2023-05-31

**Authors:** Kojiro Uetani, Takuya Uto

**Affiliations:** a Department of Industrial Chemistry, Faculty of Engineering, Tokyo University of Science 6-3-1 Niijuku, Katsushika-ku Tokyo 125-8585 Japan uetani@ci.tus.ac.jp; b Graduate School of Engineering, University of Miyazaki Nishi 1-1 Gakuen Kibanadai Miyazaki 889-2192 Japan t.uto@cc.miyazaki-u.ac.jp

## Abstract

Analysis of the attractive interaction between intrinsically twisted cellulose nanofibers (CNFs) is essential to control the physical properties of the higher-order structures of CNFs, such as paper and spun fiber. In this study, a finite element model reflecting the typical morphology of a twisted CNF was used to analyze the attractive interaction forces between multiple approaching CNF models. For two parallel CNF models, when one of the CNF models was rotated 90° around the long-axis direction, the twisting periods meshed, giving the maximum attraction force. Conversely, when the two CNF models were approaching diagonally, the CNF models were closest at an angle of −3.2° (*i.e.*, in left-handed chirality) to give the most stable structure owing to the right-handed twist of the CNF models themselves. Furthermore, the two nematic layers were closest when one nematic layer was approached at an angle of −2° (*i.e.*, in left-handed accumulation chirality), resulting in the greatest attraction. The results characterize the unique distribution of the attractive interaction forces between twisted CNF models, and they underscore the importance of chiral management in CNF aggregates, especially intermeshing of twists.

## Abstract 2

Torsional intermeshing intensifies the attractive interactions between the finite element models of twisted cellulose nanofibers.

## Graphical abstract

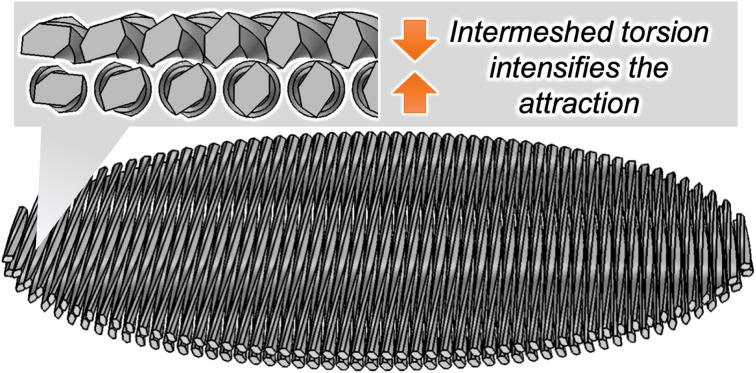

## 1. Introduction

A material composed of accumulated plant fibers that self-agglutinate by hydrogen bonding and dispersion attraction is generally defined as paper. The physical properties of paper, such as the mechanical strength, are largely dependent on the attractive interaction (*i.e.*, the adhesive force) between the fibers, in addition to shape factors, such as the length and thickness of the constituent fibers. For example, micron-size pulp fibers are generally paper made through beating and refining, which adjusts the accumulation structure by changing the fiber morphology and controls the properties of the paper by the degree of adhesion. In other words, understanding the relationship between the accumulation structure and its attractive interaction is essential for controlling the properties of paper materials.

Cellulose nanofibers (CNFs) obtained by nanofibrillation of wooden pulp fibers can be filtered and dried in the same way as pulp fibers to give paper.^1^ A wooden CNF is a crystalline fiber consisting of approximately 18 linear cellulose molecular chains,^2,3^ and unlike pulp fibers, it must be used without artificially changing its morphology (swelling or dissolving) so as to not destroy the high functionality of its extended-chain crystal structure. Therefore, the morphology of CNFs, and the attractive interaction, which depends on their accumulation structure, are directly related to the physical properties of the nanopaper. Although the resultant structures that can experimentally form (random, partial nematic, or uniaxially aligned structures) have been investigated,^4,5^ few systematic analyses of the interaction forces, which vary with the accumulation mode, have been performed.

One of the factors that makes analysis of the interaction forces between CNFs difficult is the twisted morphology of CNFs. Fibrillated single CNFs exhibit a gentle right-handed twist,^6–8^ and wooden CNFs have been reported to have a twisting period of ∼232 nm.^9^ The twisting period can be varied by drying the CNFs on a flat substrate^7^ or by changing the surface charge density.^10^ Toward characterizing the interaction forces in complex CNF aggregates, it is important to first understand the interaction forces that arise when two structural models of typical twisted CNFs approach each other. However, there has been little analysis of the interaction forces considering the twisted morphology of CNFs, either experimentally or computationally.

Cellulose nanocrystals (CNCs), highly crystalline needle-like short fibers, are produced by acid hydrolysis of plant pulp to remove amorphous parts. When twisted CNFs or CNCs concentrate in water, they self-organize into cholesteric liquid-crystal structures with helically stacked aligned layers.^11,12^ Drying of this liquid crystal narrows the nematic layer spacing in the thickness direction, resulting in a paper that maintains partial chirality.^13^ The formation principle of cholesteric liquid crystals has been characterized by the entropy,^14,15^ crystallite bundles for CNCs,^16^ steric and electrostatic interactions between chiral viruses,^17^ and screw-like rod threads engaging with each other,^14,18^ but there have been few analyses of the short-range attractive interaction energy assuming dense CNF films after removing/drying the dispersion medium. Films made of CNFs or CNCs with a nematic-ordered structure have been reported to have better physical properties, such as higher density, higher mechanical strength, higher thermal conductivity, and lower gas permeability, than films without a nematic structure.^4,19,20^ A prominent example is the better mechanical properties exhibited by Bouligand structures in biological tissues.^21–23^ However, it is very difficult to experimentally determine if the good physical properties of cholesteric structures are due to their unique fiber orientation or special attractive interaction forces based on the accumulation chirality. To characterize hierarchical fiber-accumulated CNF materials, such as cholesteric structures, toward pioneering control guidelines for physical properties, it is important to elucidate the interaction force distributions for structures in which multiple twisted CNFs are oriented or close to each other at various angles.

The objective of this study is to characterize bundling and cholesteric accumulation structures in terms of the interaction energies based on the geometry of the twisted CNFs. A finite element model of a twisted CNF was used to design an arbitrary CNF accumulation structure and calculate the total interaction energy. First, the bundling structure of two twisted CNF models was analyzed to determine the distribution of interaction forces exerted by the twisted geometry. Two circular nematic layers composed of multiple parallel twisted CNF models were then modeled, and the energies when the layers were approached at different angles were calculated. The simulations demonstrated that large accumulation stabilizing ability is only exhibited when the twisted CNF models form a specific left-handed chiral configuration.

## 2. Methods

The COMSOL model (COMSOL Multiphysics 5.5, COMSOL Inc., Stockholm, Sweden) reported in a previous study^24^ was used as the finite element model for the wooden twisted CNF. In brief, the hexagonal cross section of the 18-chained CNF model and its dimensions^2^ were formed on the *yz* plane and swept for a 360° right-handed twist for a sweep length of 232 nm^9^ in the *x*-axis direction. A tetrahedral physics-controlled mesh (the finest mesh automatically set by COMSOL, resulting mesh size of 0.0465–4.65 nm) was used.

**Fig. 1:**
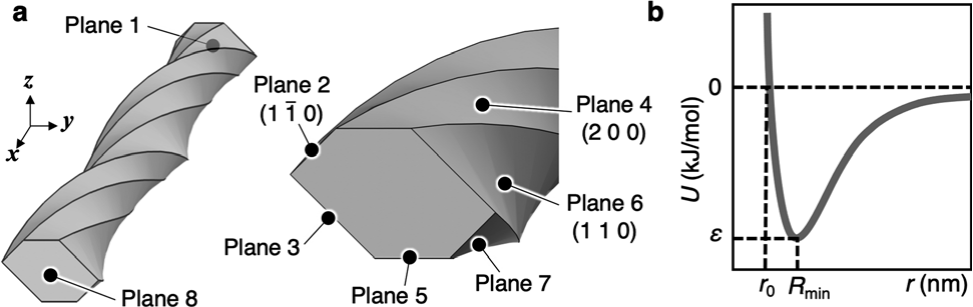
Finite element model of a twisted CNF and its parameterization. (a) Definition of the surface planes and axes of the CNF model. The definition of the axes was unified for all subsequent analyses. (b) Parameters set in the interaction energy potential functions working between the nodes of different CNF models.

The surface planes of the CNF model surface corresponded to the CNF crystal planes, as shown in Fig. 1a. The interaction energy (*U*) between the nodes in each plane was defined by the shifted-force-type Lennard-Jones 12-6 potential function:1
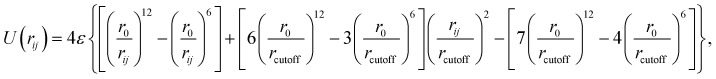
where *r*_*ij*_ is the distance between nodes *i* and *j* on different planes. The Lennard-Jones parameters, *ε* and *r*_0_, represent the potential depth and collision diameter,^25^ respectively (*U* = 0 when *r*_*ij*_ = *r*_0_). When *r*_*ij*_ is greater than the threshold (cutoff distance *r*_cutoff_), *U* is set to not be evaluated because the influence is negligible. This process is easy to implement, and it has often been used in molecular dynamics calculations because it reduces the potential energy (PE) computation time.^26^ In particular, by using a shifted-force-type potential function, *U* smoothly asymptotes to 0 at *r*_cutoff_, eliminating the discontinuous energy profile. In general, *r*_cutoff_ is set to be several times larger than *r*_0_.^26^ In this study, *r*_cutoff_ = 3.2 nm, corresponding to 6*r*_0_, was used. The fiber-to-fiber interaction energy (PE) was evaluated as the sum of *U* between contact points in the entire system.2
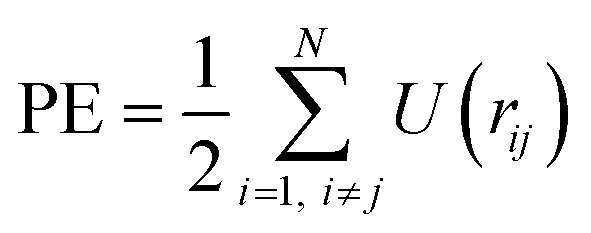


Differentiating eqn (1) with respect *r*_*ij*_ gives the force (*F*) acting between the nodes (assuming that the repulsive force is positive):3
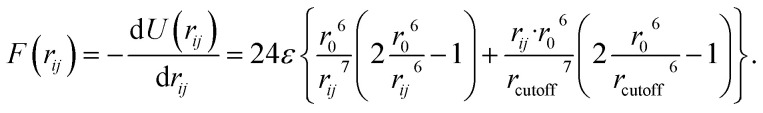
In this case, *F* = 0 at the equilibrium distance between the nodes (*R*_min_) given by

4 *R*_min_ ≈ 2^1/6^*r*_0_.

Because *r*_cutoff_ = 6*r*_0_, the second term in eqn (3) can be ignored. Substituting *r*_*ij*_ = *R*_min_ into the second derivative of eqn (1) gives a positive value, confirming that *U* has the minimum value at *R*_min_ and is in an energetically stable state. Therefore, this *R*_min_ is in good agreement with the lattice constant of the material. *R*_min_, which gives the potential minimum point of *U*, was reasonably considered to be the distance between the crystal planes, and it was set for each face of the CNF model with reference to the crystal–structure parameters (Table 1). The spacing of the crystalline planes of cellulose Iβ^27^ was used for *R*_min_ between the same crystalline planes. *R*_min_ between dissimilar crystal planes was estimated by the additive average of each crystal-plane spacing (Lorentz–Berthelot combining rule). In the actual PE calculations, these *R*_min_ values were converted to *r*_0_ by eqn (4) (Table 1). There is no information about the crystal-plane spacing for *R*_min_ involving planes 1 and 8. Therefore, for the combination of plane 1 or 8 and hydrophilic plane 2, 3, 6, or 7, the average of the *R*_min_ values for (1 1 0) × (1 1 0), (1 1̄ 0) × (1 1̄ 0), and (1 1 0) × (1 1̄ 0) was used. For the combination of plane 1 or 8 and hydrophobic planes 4 and 5, the average of the *R*_min_ values for (1 1 0) × (2 0 0) and (1 1̄ 0) × (2 0 0) was used.

**Table 1:** Potential parameter settings for the twisted CNF models

Combination	*ε* (kJ mol^−1^)	*r*_0_ (nm)	*R*_min_ (nm)
(1 1 0) × (1 1 0)	25.0	0.47	0.53
(1 1̄ 0) × (1 1̄ 0)	25.0	0.53	0.60
(1 1 0) × (1 1̄ 0)	25.0	0.50	0.565^a^
(2 0 0) × (2 0 0)	75.0	0.35	0.39
(1 1 0) × (2 0 0)	1.0	0.41	0.46^b^
(1 1̄ 0) × (2 0 0)	1.0	0.44	0.495^c^

a (0.53 + 0.60)/2 = 0.565.

b (0.53 + 0.39)/2 = 0.46.

c (0.60 + 0.39)/2 = 0.495.

When eqn (1) and (4) are coupled, *U*(*R*_min_) ≈ −*ε*. Because *U* → 0 at the dissociation limit *r*_*ij*_ → *r*_cutoff_, *ε* corresponds to the binding energy between the nodes. The values of *ε* were taken from the parameters applied in coarse-grained CNF models at similar scales, taking into account the mesh size.^28^ Note that in this coarse-grained CNF model, it has been reported that the binding energy between two CNFs roughly reproduces the value evaluated by the all-atom model, so the parameters are thought to be highly reliable. By treating (1 1 0) and (1 1̄ 0) as equivalent planes, *ε* was determined to be 25, 75, and 1.0 kJ mol^−1^ for the combinations of hydrophilic/hydrophilic, hydrophobic/hydrophobic, and hydrophilic/hydrophobic surfaces, respectively. In this study, planes 2, 3, 6, and 7 were defined as hydrophilic surfaces and planes 4 and 5 were defined as hydrophobic surfaces. Planes 1 and 8 were also treated as hydrophilic surfaces, with *ε* depending on the combination of the constituent surfaces (Tables S1 and S2†).

*D* denotes the distance between the centers of gravity of the CNF models/groups, and *D* in the *x*, *y*, and *z* directions is denoted *D*_*x*_, *D*_*y*_, and *D*_*z*_, respectively. Min. PE represents the minimum PE.

**Fig. 2:**
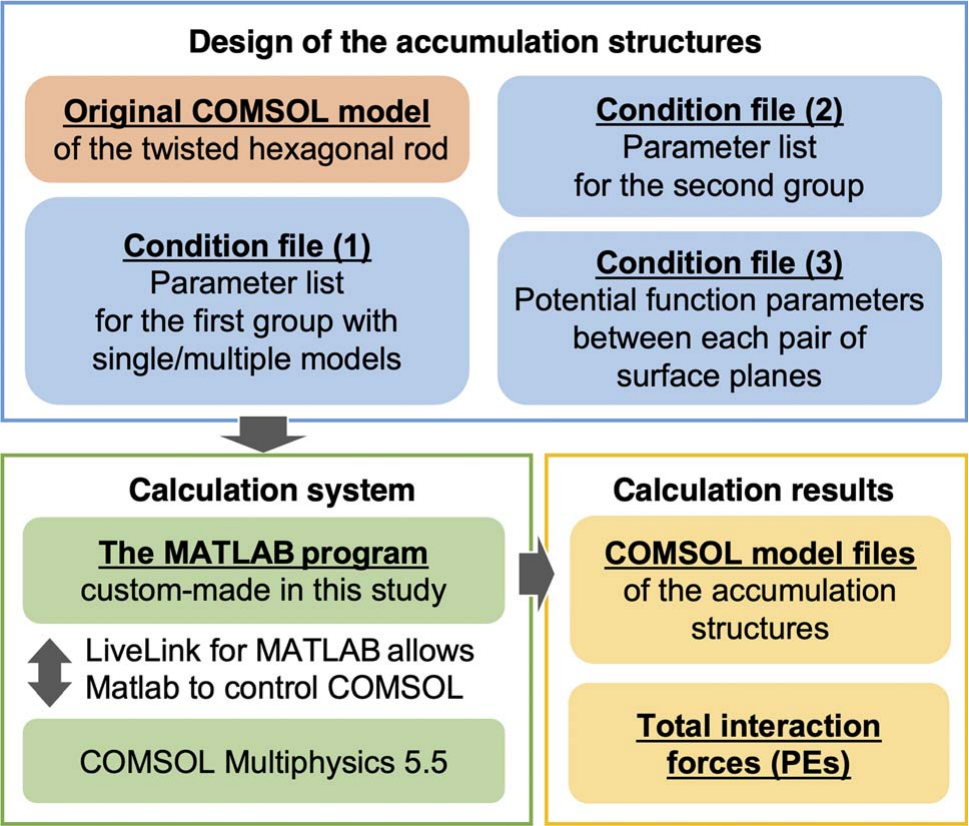
Relationship diagram of the system for designing the accumulation structures of twisted hexagonal CNF models and calculating their PEs.

For several CNF models in close proximity, the potential energy (*U*) between the nodes of each CNF model was summed to estimate the total interaction force (PE) in the accumulated structure. First, as shown in Fig. 2, condition (1) established the basic form of the CNF model and the first group with one or more CNF models. Condition (1) includes the coordinates of the center of gravity (*x*, *y*, *z*) of the model end face, the sweep length of the *yz* cross section in the *x* direction, the twist angle during the sweep, and the rotation angle around the sweep axis (*x* axis). The *y* and *z* axes pass through the center of gravity of the model/group. Next, condition (2) duplicates the first group. The parallel shift distances in the *x*, *y*, and *z* directions and the rotation angles around the *x*, *y*, and *z* axes passing through the center of gravity of the model/group are then set for the second group when both groups are placed close to each other. In other words, the accumulated structures that can be analyzed by this system are limited to relatively simple structures that duplicate the first group and adjust its arrangement. Finally, as condition (3), we used the potential functions and parameters set in eqn (1) and Table 1 (more specifically, Tables S1 and S2† for *ε* and *r*_0_). These conditions were loaded into a custom-made Matlab program commissioned to the KOBELCO Research Institute, Inc. (Kobe, Japan) to control COMSOL using LiveLink for Matlab (COMSOL Inc., Stockholm, Sweden). The total interaction forces (PEs) of the final accumulated structures were calculated with this program.

## 3. Results and discussion

### 3.1 Operation verification of the calculation system

**Fig. 3:**
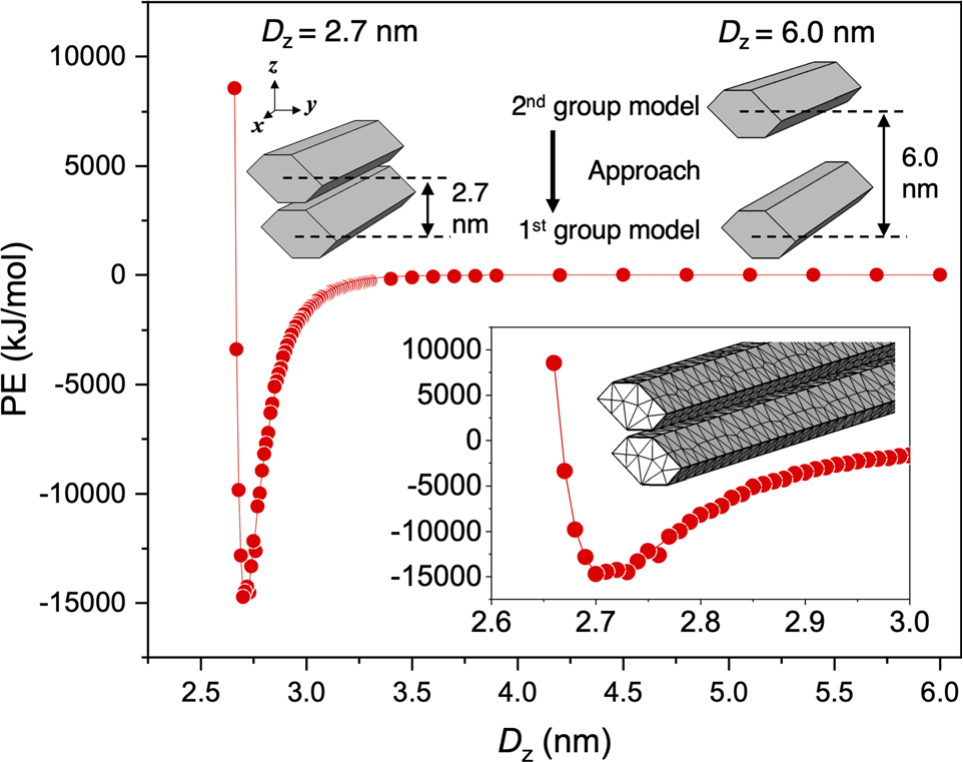
Distance between the centers of gravity in the *z* direction (*D*_*z*_) and PE results to validate the computational system with CNF models without twists. The insert shows an enlarged plot around Min. PE and the mesh structure of the CNF models.

To verify that the constructed calculation system worked as configured, the approach behavior of two hexagonal prism CNF models with torsion removed from the CNF models was calculated. Planes 4 and 5 of the CNF models were set to approach each other with the parameters and settings in Table 1, and *ε* for all other planes was set to 0. The results of PE calculations for different distances between the centers of gravity of the two CNF models are shown in Fig. 3. PE, which was 0 kJ mol^−1^ at long distance, became negative as the distance decreased, indicating an attractive interaction between the CNF models. As the planes further approached each other, the repulsive force rapidly increased and diverged, giving a Lennard-Jones-type potential curve.

For this CNF model, the PE plot tended to be somewhat scattered owing to the close intermodel distance relative to the nodal spacing and the irregular placement of the nodes. Therefore, to precisely calculate the Min. PE, OriginPro 2020 and 2021 (OriginLab Corp., MA, USA) were used to fit the PE plot (Fig. 3) according to5
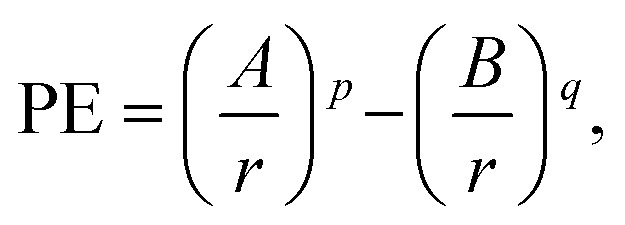
where *A*, *B*, *p*, and *q* are constants. The fitted functions were analyzed by Wolfram Alpha Pro (Wolfram Research, Champaign, IL, USA) to obtain the Min. PE and its *D*_*z*_. This analytical approach was implemented for all of the subsequent calculations. In rare cases, the CNF models were too close together and overlapped (the system identified the overlap and stopped the calculation), resulting in the plots not reaching a local minimum PE, in which case the smallest PE among the results was used as Min. PE.

The CNF model in Fig. 3 without torsion gave PE = −14 921 kJ mol^−1^ at *D*_*z*_ = 2.71 nm. Because the thickness of the CNF model in the *z* direction was set to 2.34 nm, the distance between the CNF model surfaces for Min. PE was calculated to be 0.37 nm. This distance was close to the *R*_min_ value (0.39 nm) set in Table 1. *r*_cutoff_ was set to 3.2 nm, so the PE was 0 when *D*_*z*_ was greater than 5.54 nm. The above results reproduced the set parameters, confirming that the system can correctly perform the calculations.

### 3.2 Interaction force between two parallel twisting CNF models

**Fig. 4:**
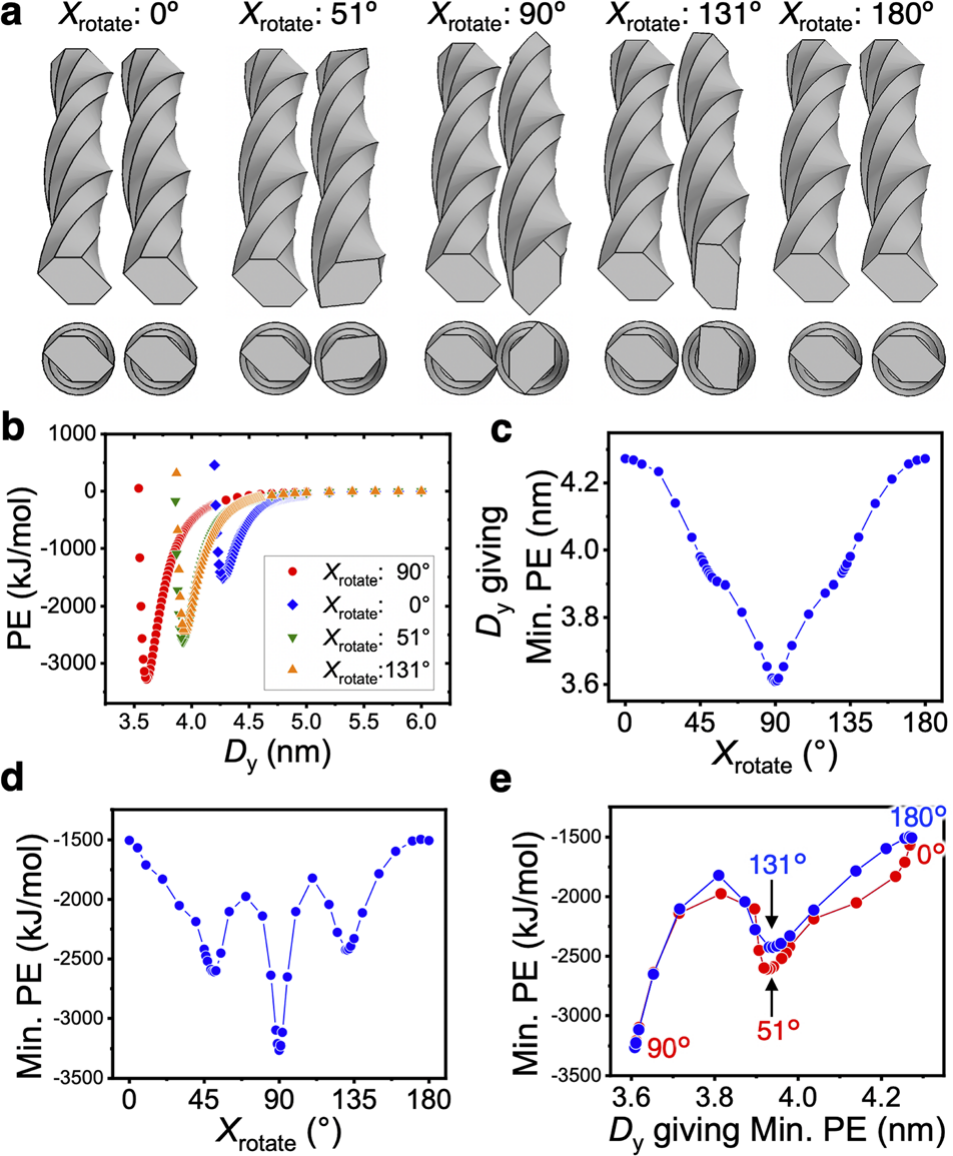
Interaction-force distribution between two twisted CNF models adjacent in the *y*-axis direction. (a) Arrangements giving Min. PE when the first CNF model on the left was fixed and the second CNF model on the right was rotated around the *x* axis (rotation angle *X*_rotate_). (b) Distance dependence of PE at each *X*_rotate_. Distributions of (c) *D*_*y*_ giving Min. PE and (d) Min. PE against *X*_rotate_. (e) Relationship between *D*_*y*_ giving Min. PE and Min. PE.

To investigate the relationship between the bundle structure of the two 232 nm-long twisted CNF models in Fig. 1 and their PE, the first CNF model on the left-hand side was fixed and the parallel second CNF model on the right-hand side (adjacent in the *y*-axis direction) was rotated around the *x* axis (long axis). The relationship between Min. PE and the distance *D*_*y*_ giving Min. PE was analyzed with respect to the rotation angle around the *x* axis (*X*_rotate_) (Fig. 4).

*D*_*y*_ was largest for *X*_rotate_ = 0° and 180°, and it was smallest for *X*_rotate_ = 90°. When *X*_rotate_ = 90°, the twisting periods of the left and right CNF models meshed and the structure allowed the CNF models to be closest (Fig. 4a). In this case, Min. PE was −3264 kJ mol^−1^, which was approximately twice the value for *X*_rotate_ = 0°, because the area in which the models could interact was maximized. For all of the *X*_rotate_ values, the dependence of *D*_*y*_ on PE resulted in smooth profiles (Fig. 4b), unlike the case of the untwisted CNF model in Fig. 3. The distribution of *D*_*y*_ from *X*_rotate_ = 0° to 180° was symmetrical around 90° (Fig. 4c). The distribution of Min. PE was also highly symmetric with respect to *X*_rotate_ (Fig. 4d). The local minima at ∼51° and ∼131° suggest the presence of a new metastable association structure. The nonlinear Min. PE distribution is considered to be because of the different parameters defined for each surface plane.

The interaction force distribution of the two parallel CNF models was exactly the same when the second CNF model was placed above the first CNF model (adjacent in the *z*-axis direction), as shown in Fig. S1.† When the CNF models were placed parallel to each other in the *z*-axis direction, the smallest PE was obtained for *X*_rotate_ = 90°. From this result, it can be concluded that the interaction force was correctly calculated regardless of the relative positions of the CNF models.

### 3.3 Most stable accumulation structure for two twisted CNF models with 90° difference in *X*_rotate_

In Section 3.2, the first CNF model was fixed and only *X*_rotate_ of the second CNF model was varied, but a variety of relative configurations with a 90° difference in *X*_rotate_ between the CNF models can be taken. Therefore, we searched for the most stable structure by rotating the first CNF model around the *x* axis while maintaining a 90° difference in *X*_rotate_ with the second CNF model.

**Fig. 5:**
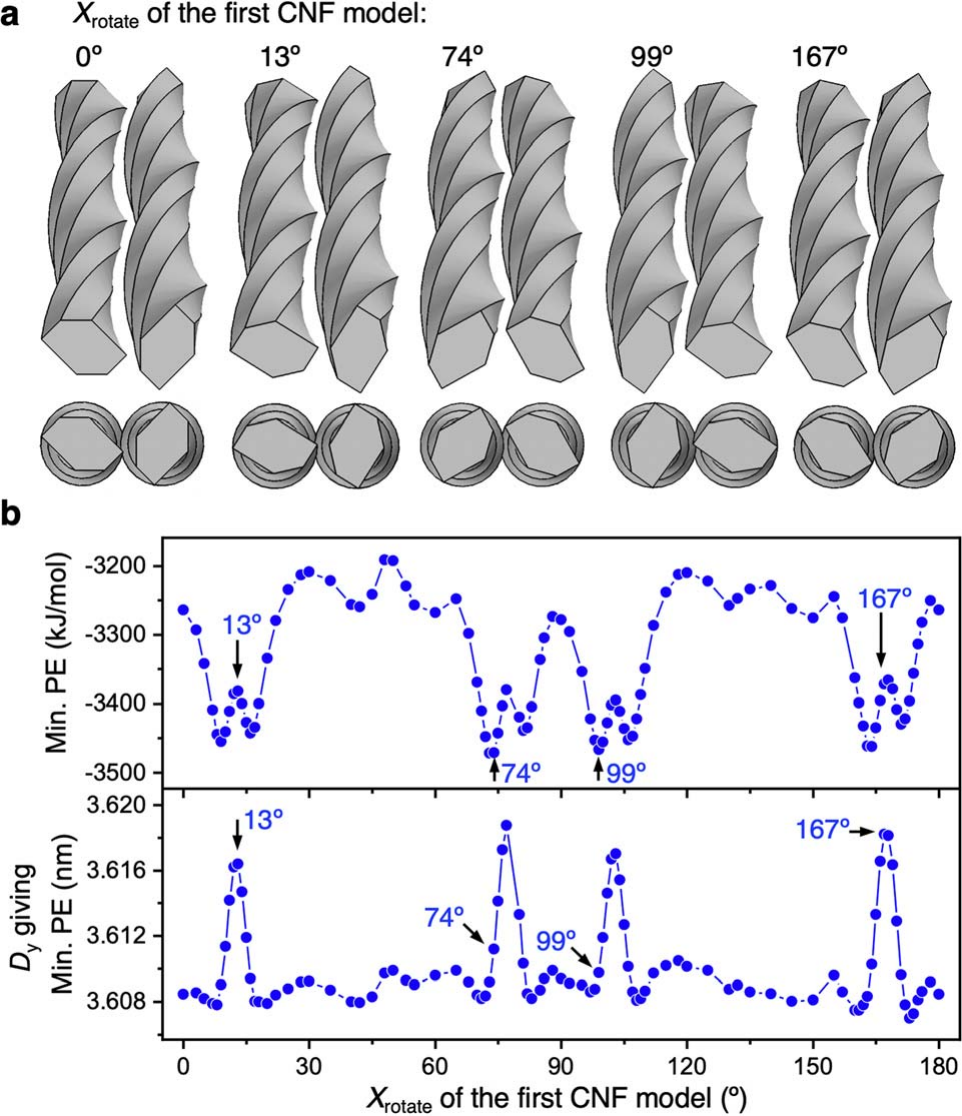
Interaction-force distribution between two parallel CNF models with 90° different *X*_rotate_. (a) Arrangements giving Min. PE when *X*_rotate_ of the second CNF model on the right remained 90° more than that of the first CNF model on the left and the first CNF model was rotated around the *x* axis. (b) Distributions of Min. PE and *D*_*y*_ against *X*_rotate_ of the first CNF model.

The structure was constructed by varying *X*_rotate_ of the first CNF model and setting the *x*-axis rotation angle of the second model to *X*_rotate_ + 90°, as shown in Fig. 5a. In all cases, the torsion period of the CNF model was engaged, indicating that the CNF models were close together and the interaction forces were high. By plotting Min. PE and *D*_*y*_ against *X*_rotate_ for the first CNF model (Fig. 5b), we found that overall *D*_*y*_ was small and Min. PE (absolute value) was at a large level. However, they showed periodic fluctuations. Interestingly, at *X*_rotate_ when *D*_*y*_ was temporarily large, the absolute values of Min. PE became larger in the vicinity (approximately −3400 kJ mol^−1^ or greater). The relationship between Min. PE and *D*_*y*_ was clearly different at small *D*_*y*_ (approximately 3.61 nm or less) and at larger *D*_*y*_ (Fig. S2†). From the hexagonal edge lengths of the CNF cross section, it was inferred that the periodic appearance of surfaces with different widths temporarily increased the number of nodes that can interact, lowering Min. PE. Furthermore, a temporary increase in *D*_*y*_ and a temporary decrease in Min. PE (absolute value) occurred at the same *X*_rotate_ (Fig. 5b). It is considered that an increase in *D*_*y*_ would increase the distance between the CNF models, as a tradeoff for an increase in the interaction area, resulting in a slight decrease in Min. PE.

### 3.4 Diagonal proximity and interaction forces of two twisted CNF models

Min. PE and *D*_*z*_ with respect to the rotation angle *Z*_rotate_ were evaluated when the centers of gravity of CNF models with *X*_rotate_ = 0° were placed on the same *z* axis and the first model (bottom side) was fixed and the second CNF model (top side) was rotated around the *z* axis with the center of gravity.

**Fig. 6:**
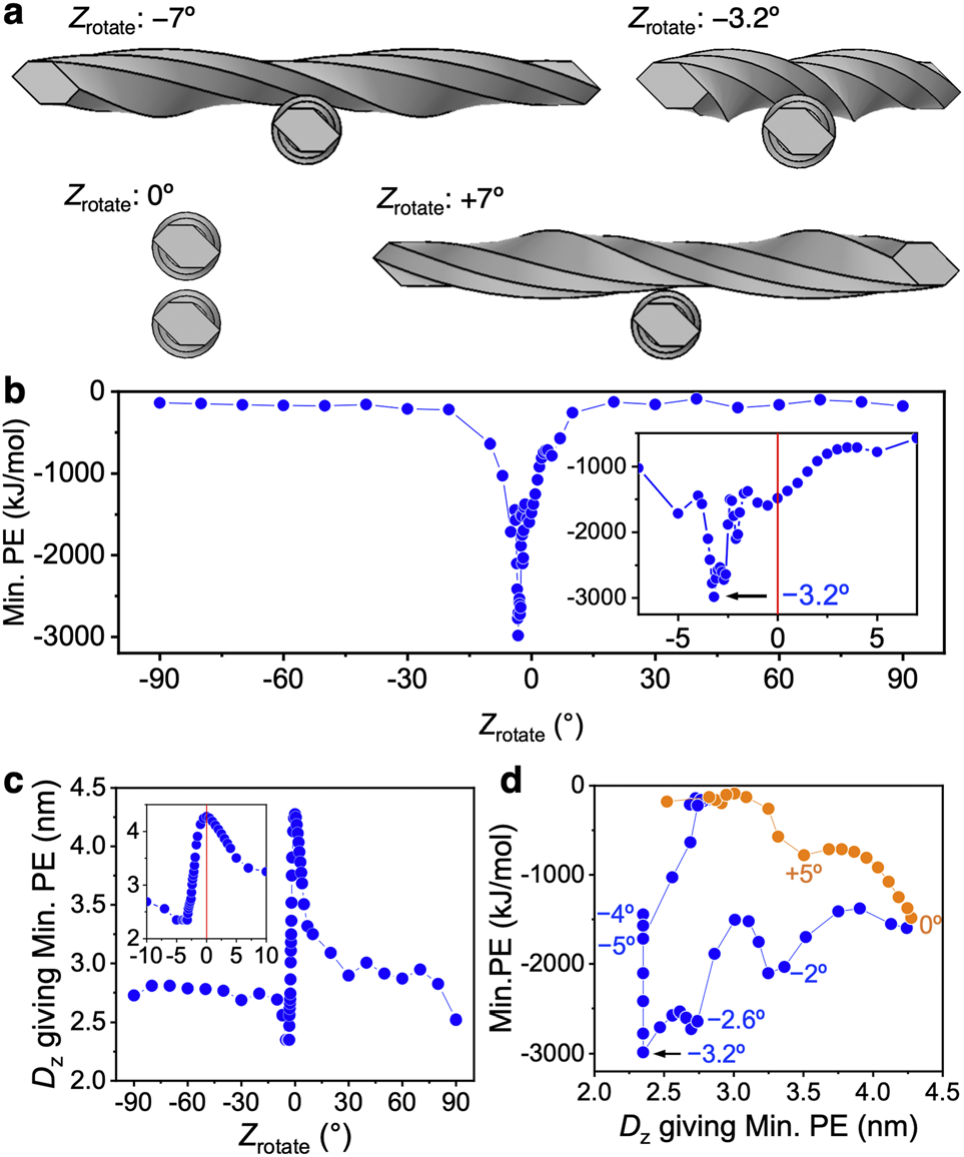
Interaction force distribution between CNF models rotating about the *z* axis. (a) Arrangement giving Min. PE at each *Z*_rotate_. (b) Min. PE distribution relative to *Z*_rotate_. (c) Distribution of *D*_*z*_ that gives Min. PE against *Z*_rotate_. (d) Relationship between *D*_*z*_ giving Min. PE and Min. PE.

When the 232 nm-long right-handed-twist CNF model approached at an angle, the direction of the twist directly affected the approach distance (Fig. 6a). For large positive and negative *Z*_rotate_ values (absolute values greater than ∼7°), the contact area was small and the interaction force was weak (Fig. 6b). Conversely, when the absolute value of *Z*_rotate_ was small, the asymmetry caused by the twisting direction of the CNF model was clearly expressed.

When *Z*_rotate_ was negative, the chirality of the two-model configuration was left-handed, showing a maximum Min. PE (absolute value) of −2987 kJ mol^−1^ at *Z*_rotate_ = −3.2°. This local attraction was approximately 15 times larger than that at *Z*_rotate_ = ±(30–90)°. In particular, when *Z*_rotate_ was approximately −2.6° to −3.3°, the right-handed twists of the CNF models meshed with each other, the CNF models more closely approached (*D*_*z*_ became smaller), and their Min. PE became smaller than −2500 kJ mol^−1^ (Fig. 6c and d). We confirm that the accumulation mode of the twisted CNF model is consistent with the prediction derived from entropy^14,18^ that screw-like rods pack tightly by interlocking threads along grooves.

For calculations with *Z*_rotate_ = −3.2° to −5°, as *D*_*z*_ decreased, the CNF models overlapped before the PE reached the local minimum PE, making calculations with small *D*_*z*_ impossible. Therefore, the smallest PE value in the calculated range was used, and at that time the *D*_*z*_ values were all 2.35 nm (Fig. 6c). When *Z*_rotate_ = −3.2° to −5°, planes 4 and 5 defined in Fig. 1 are considered to be mainly approaching each other. Taking into account the *z*-direction thickness of the CNF model of 2.34 nm, the distance between the surfaces approaches a distance below *R*_min_, so the occurrence of local repulsion can be inferred. Nevertheless, the attractive Min. PEs for the entire system were still dominant at *Z*_rotate_ = −3.2° to −5°. We speculate that in oblique approach at the small and negative *Z*_rotate_, the various surface and nodal combination are at the distances that exhibit attractive interactions.

When *Z*_rotate_ was positive, *D*_*z*_ tended to be larger because the right-handed twists of the CNF models did not engage and the convexities were closer together (Fig. 6c). The resulting Min. PE (absolute value) was smaller and the Min. PE distribution was completely asymmetric with respect to the negative *Z*_rotate_ case (Fig. 6b and d). This result was the opposite of the parallel CNF model shown in Fig. 4 and 5.

### 3.5 Effect of *X*_rotate_ at *Z*_rotate_ = −3.2°

**Fig. 7:**
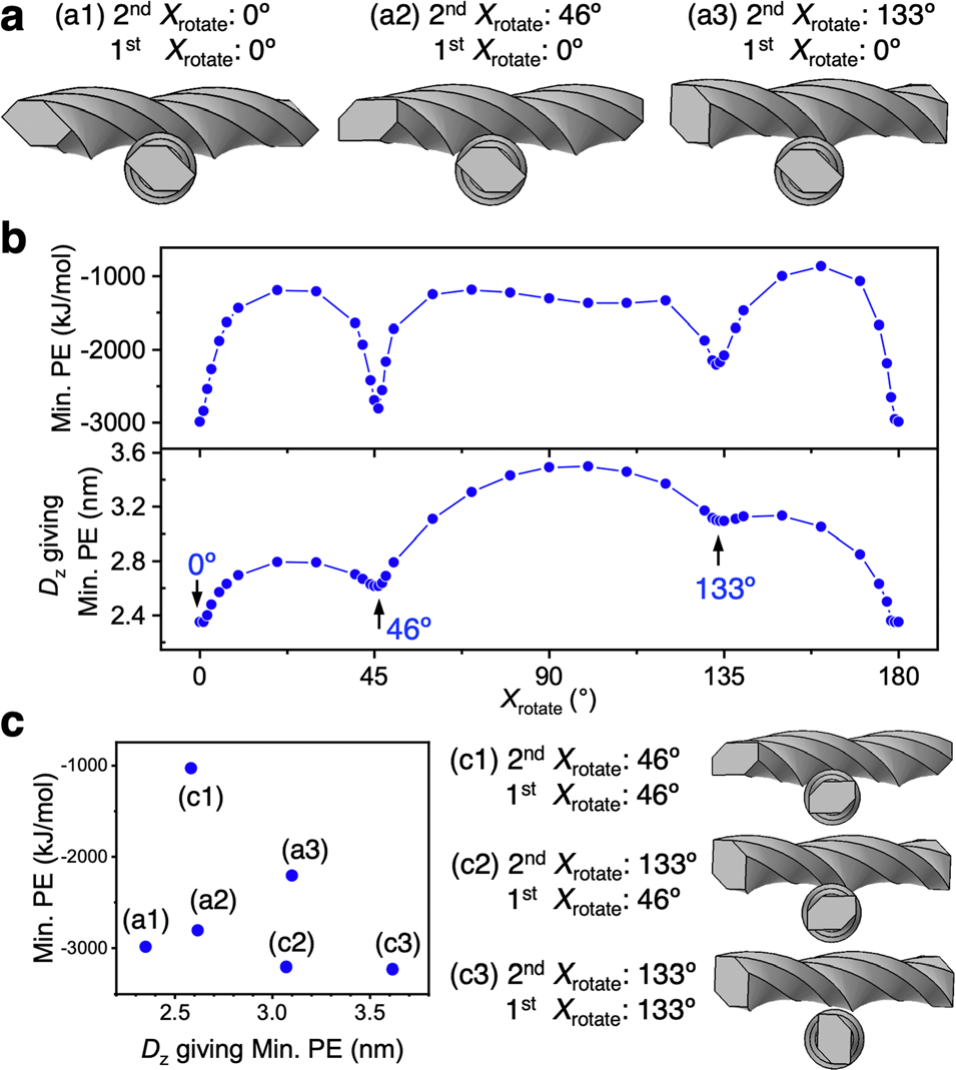
Min. PE search by fixing *Z*_rotate_ = −3.2° for the upper CNF model and varying *X*_rotate_. (a) Arrangements giving Min. PE. (b) Distribution of Min. PE and *D*_*z*_ with respect to *X*_rotate_ of the second CNF model when *X*_rotate_ = 0° for the first CNF model. (c) Min. PE and *D*_*z*_ relationships for six different structures with *X*_rotate_ = 0°, 46°, and 133° for both CNF models.

Within the oblique approach of the two CNF models, *Z*_rotate_ of the second CNF model was fixed at −3.2° and the Min. PE distribution was evaluated by varying *X*_rotate_ of the second CNF model, while that of first CNF model was fixed at 0°. Local minima of Min. PE occurred when *X*_rotate_ of the second CNF model was 0°, 46°, and 133° (Fig. 7). In the lower first CNF model, plane 5 was mainly in close proximity to the upper CNF model near the center of gravity. Conversely, the upper second CNF model was closest to the lower CNF model mainly for plane 4 when *X*_rotate_ = 0°, for plane 6 when *X*_rotate_ = 46°, and for plane 7 when *X*_rotate_ = 133°. From Tables 1, S1, and S2,† the combination of planes 4 and 5 (when *X*_rotate_ = 0°) has the largest *ε* and smallest *R*_min_ (*r*_0_), and the result is reasonable given the largest Min. PE (absolute value) in Fig. 7. The combinations of planes 5 and 6, and 5–7 (*X*_rotate_ = 46° and 133°, respectively) both have the smallest *ε*, and *R*_min_ (*r*_0_) is smaller for planes 5 and 6. The effect is directly seen in the smaller *D*_*z*_ for *X*_rotate_ = 46° in Fig. 7b. Although the interaction forces owing to other surfaces are of course included, it is clearly shown that the interaction force was the largest when the approaching surfaces were parallel to each other.

Changing *X*_rotate_ of the first CNF model, which was fixed at 0°, 46°, and 133°, was considered to change the proximity plane to the second CNF model. Therefore, we investigated Min. PE for six combinations of *X*_rotate_ ((a1)–(a3) in Fig. 7a and (c1)–(c3) in Fig. 7c). The structure (c3) became stable with the largest Min. PE (absolute value), while (c1) became unstable with the smallest Min. PE (Fig. 7c). In structure (c1), planes 3 and 6 were the closest to each other, and in (c3), planes 2 and 7 were the closest to each other, which both correspond to combinations of hydrophilic surfaces of the CNF. The intermodel distance for (c1) was close with *D*_*z*_ = ∼2.6 nm, while *D*_*z*_ for (c3) was the largest among the six CNF models (*D*_*z*_ = ∼3.6 nm).

The maximum interaction force at *Z*_rotate_ = −3.2° for the second CNF model was −3230 kJ mol^−1^, as indicated by only (c3). In Fig. 7b, Min. PEs above −2000 kJ mol^−1^ were obtained at many *X*_rotate_ values, and only the limited structures had more stable Min. PE. On the other hand, in Fig. 5, most structures showed −3200 to −3500 kJ mol^−1^ regardless of the *X*_rotate_ value, indicating that the parallel bundling at *X*_rotate_ = 90° (at *Z*_rotate_ = 0°) is more stable than the diagonal approach in Fig. 7 for two twisted CNF models above this length (≧232 nm).

### 3.6 Most stable structure with two nematic layers

**Fig. 8:**
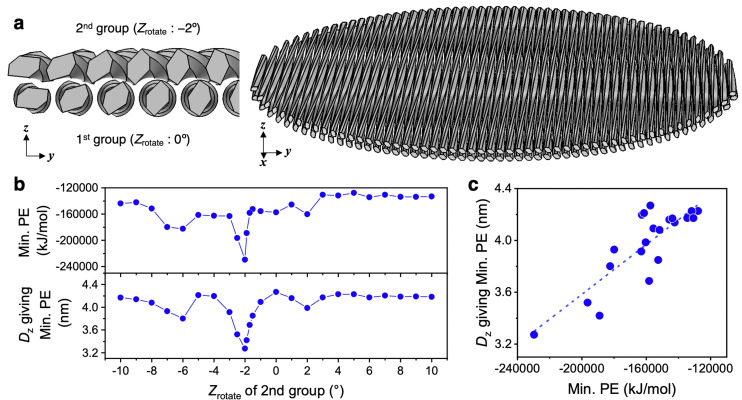
Min. PE search for two nematic layers in proximity. (a) Structure giving Min. PE when the number of torsional CNF models in one group was 54 and the distance between the CNF models in the *y*-axis direction was 4.27253 nm and the upper second group was rotated at *Z*_rotate_ = −2°. (b) Min. PE and *D*_*z*_ distributions relative to *Z*_rotate_ of the second group. (c) Relationship between Min. PE and *D*_*z*_.

Two circular nematic layers with multiple twisted CNF models aligned in parallel were modeled to evaluate the interaction energy when the layers approached at different rotation angles. Within the nematic layer, *n* CNF models were arranged in a circular shape with a diameter of 232 nm, parallel to the *x* axis and lineally symmetric with the *x* axis as the axis of symmetry to preserve the rotational symmetry around the *z* axis (the detailed design is described in Appendix 1). The twisted CNF models were placed in a single nematic layer with the same relative arrangement as in Fig. 4a with *X*_rotate_ = 0°. *D*_*y*_ = 4.27253 nm, which gives Min. PE, was used, and the resulting structure with 54 CNF models circularly arranged in one nematic layer was designed (Fig. 8a). The first layer (the first group) was duplicated in the *z*-axis direction to form the second group, and Min. PE and *D*_*z*_ (in this case, the distance between the centers of gravity of each group) were calculated when both groups approached relative to the rotation angle around the *z* axis with the center of gravity of the second group (*Z*_rotate_).

The distribution of Min. PE and *D*_*z*_ was asymmetric with respect to *Z*_rotate_ = 0°, and when *Z*_rotate_ = −2° for the second group, Min. PE and *D*_*z*_ were the lowest (Fig. 8b). At *Z*_rotate_ = −2°, Min. PE was −229 660 kJ mol^−1^, which was approximately 1.6 times higher in absolute value than at positive *Z*_rotate_ (approximately −140 000 kJ mol^−1^). In the approach of the nematic layers, as in the case of the two CNF models (Fig. 6 and 7), the most stable structure was the one that accumulated in the left-handed direction.

Min. PE and *D*_*z*_ were roughly correlated with the slope of the fitted line was found to be 9.59 × 10^−6^ (nm (kJ mol^−1^)^−1^) (*R*^2^ = 0.74) (Fig. 8c), indicating that the “layer-to-layer approachability,” which depends on the twisting period of the CNF model and the intermodel distance within a nematic layer, was directly related to the interaction force. Min. PE (absolute value) was larger in the chiral integration case (*Z*_rotate_ = −2°) than in the case where the nematic layers were perfectly parallel (*Z*_rotate_ = 0°, *i.e.*, the uniaxially aligned structure). In dried films containing the nematic structure, the angle between the layers was found to play an important role in the fiber-packing structure and interaction forces.

## 4. Conclusions

A finite element model of a twisted CNF has been used to investigate the relationship between the accumulation structure and the attractive interaction. For two parallel CNF models with the long axis along the *x* axis, when one of the CNF models had *X*_rotate_ = 90°, the torsion periods intermeshed and showed twice the attraction force when *X*_rotate_ = 0°. Conversely, when the two CNF models approached diagonally (rotated around the *z* axis), Min. PE was asymmetric with respect to positive and negative *Z*_rotate_, and it was most stable at negative *Z*_rotate_ (−3.2° to the left), showing 15 times larger attraction force than at larger angles. This tendency for left-handed accumulation chirality was also found for the attraction force in the proximity of two nematic layers, and it was found that the layers were the closest when the *Z*_rotate_ of the second layer was −2°, giving 1.6 times larger attraction force than at positive *Z*_rotate_.

The interaction calculation using the finite element twisted CNF model analyzes very short distances assuming dry conditions without a dispersion medium, ignoring the effect of moisture to which real CNFs are often exposed. However, the accumulation chirality based on the near-range attraction in the dry state elucidated in this study are well matched with the left-handed cholesteric liquid crystals of CNFs dispersed in water. In addition, compared with simple uniaxially oriented CNF films (corresponding to *Z*_rotate_ = 0° in Fig. 8), CNF films with a cholesteric structure (especially for *Z*_rotate_ = −2°) showed significantly larger total attraction energy, and thus they are expected to have higher paper strength. The results directly demonstrated that the left-handed chirality of the configuration (*i.e.*, the intermeshing of the right-handed twists) is a source of high functionality in CNF accumulations, and they confirm the importance of chiral management for improving the physical properties in terms of the attractive interaction energy.

## Author contributions

K.U.: conceptualization; data curation; funding acquisition; investigation; methodology; software; validation; visualization; writing – original draft; writing – review & editing, T.U.: formal analysis; funding acquisition; investigation; methodology; supervision; validation; writing – review & editing.

## Conflicts of interest

There are no conflicts of interest to declare.

## Supplementary Material

RA-013-D3RA01784B-s001
