# Supplementary material for: Strong attractive interaction between finite element models of twisted cellulose nanofibers by intermeshing of twists
Source: RSC Adv. 2023 May 31;13(24):16387–95. doi: 10.1039/d3ra01784b (PMC10231428; doi:10.1039/d3ra01784b)
Supplement: RA-013-D3RA01784B-s001 [file RA-013-D3RA01784B-s001.pdf]

Electronic Supplementary Material (ESI) for RSC Advances.
This journal is © The Royal Society of Chemistry 2023

Supporting Information

Strong attractive interaction between finite element models of twisted cellulose nanofibers by intermeshing of twists

Kojiro Uetani,*[a] and Takuya Uto[b]

*To whom correspondence should be addressed.

E-mail: uetani@ci.tus.ac.jp (K. Uetani), t.uto@cc.miyazaki-u.ac.jp (T. Uto)

This PDF file includes:

Tables S1 and S2

Figures S1 and S2

Appendix 1, including Figures S3 to S5

Table S1. ε values for the proximity combinations of each two planes of the twisted CNF model defined in Figure 1.

ε [kJ/mol]	Plane 1	Plane 2	Plane 3	Plane 4	Plane 5	Plane 6	Plane 7	Plane 8
**Plane 1**	25	25	25	1	1	25	25	25
**Plane 2**		25	25	1	1	25	25	25
**Plane 3**			25	1	1	25	25	25
**Plane 4**				75	75	1	1	1
**Plane 5**					75	1	1	1
**Plane 6**						25	25	25
**Plane 7**							25	25
**Plane 8**								25

Table S2. r_0 values for the proximity combinations of each two plane of the twisted CNF model defined in Figure 1.

r_0 [nm]	Plane 1	Plane 2	Plane 3	Plane 4	Plane 5	Plane 6	Plane 7	Plane 8
**Plane 1**	0.50	0.50	0.50	0.43	0.43	0.50	0.50	0.50
**Plane 2**		0.53	0.50	0.44	0.44	0.50	0.53	0.50
**Plane 3**			0.47	0.41	0.41	0.47	0.50	0.50
**Plane 4**				0.35	0.35	0.41	0.44	0.43
**Plane 5**					0.35	0.41	0.44	0.43
**Plane 6**						0.47	0.50	0.50
**Plane 7**							0.53	0.50
**Plane 8**								0.50

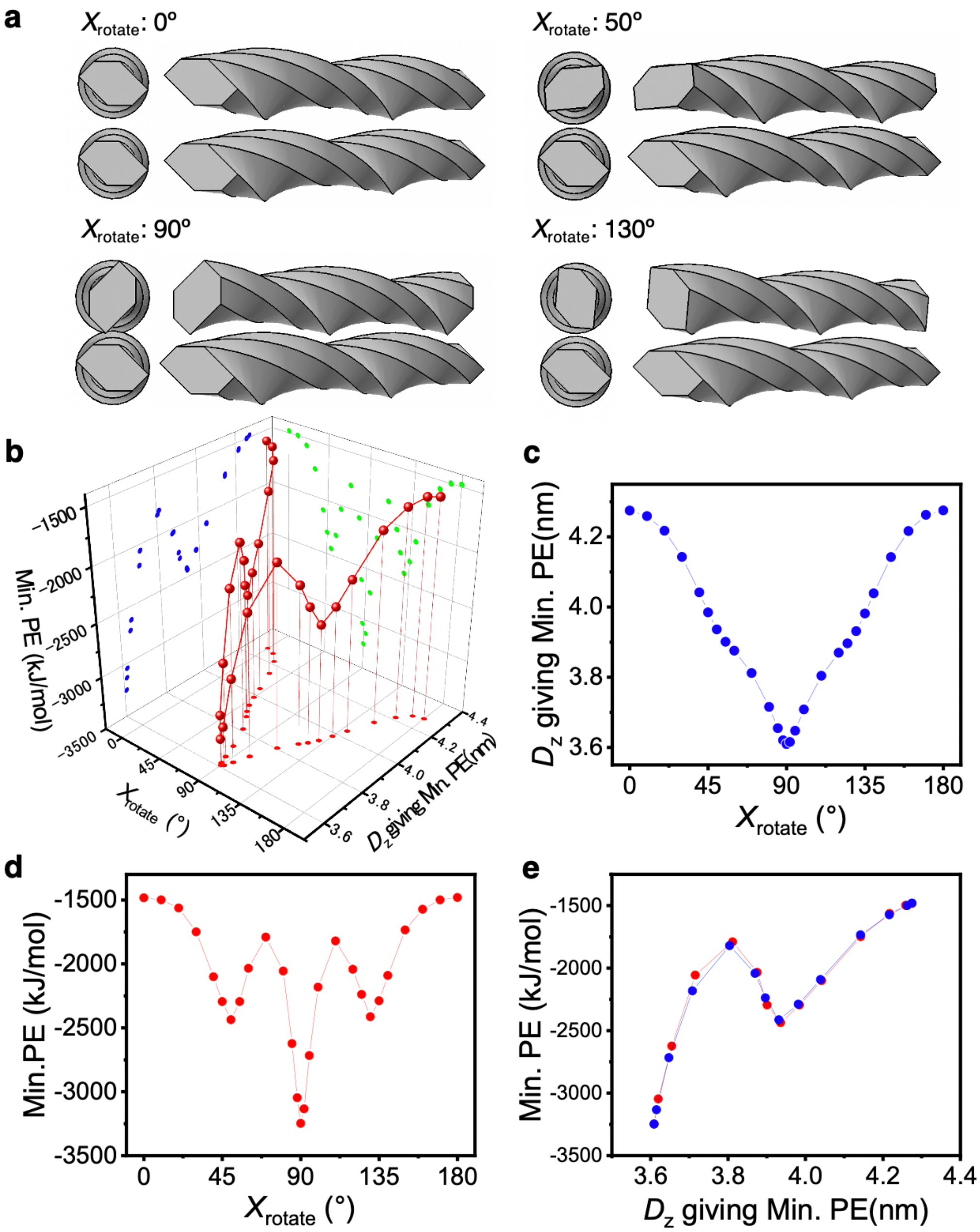

Figure S1. Interaction-force distribution between two twisted CNF models adjacent in the *z*-axis direction. (a) Arrangements giving the minimum PE (Min. PE) when the lower first CNF model was fixed and the upper second CNF model was rotated around the *x* axis at rotation angle X_{rotate}. (b) Relationship between X_{rotate}, D_z, and Min. PE. Distributions of (c) D_z giving Min. PE and (d) Min. PE against X_{rotate}. (e) Relationship between D_z giving Min. PE and Min. PE.

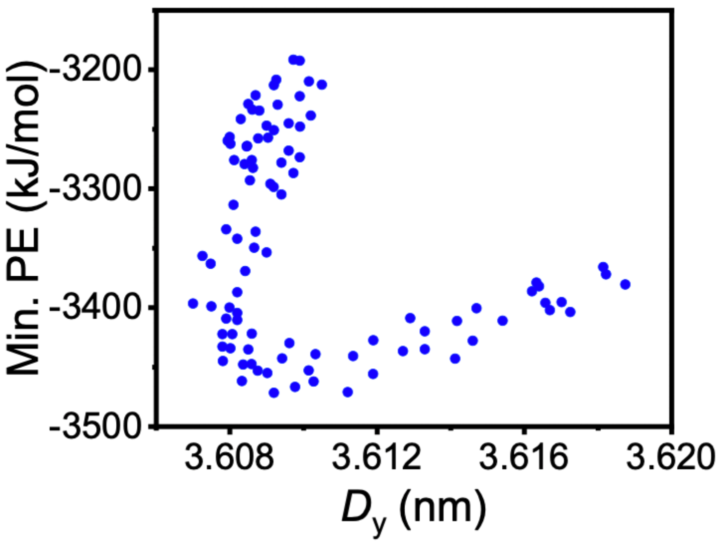

Figure S2. Relationship between Min. PE and D_y between the two parallel CNF models with 90° different X_{rotate} shown in Figure 5.

Appendix 1

The following structural design was applied to evaluate the approach distance, rotation angle, and PE of two groups of nematic layers with multiple twisted CNF models as one group. To maintain rotational symmetry around the z axis within the nematic layer, n CNF models were arranged in a circular shape with a diameter of 232 nm (corresponding to one twist cycle of a CNF model), parallel to the x axis and lineally symmetric with the x axis as the axis of symmetry. The coordinate settings for the CNF model comprising the first CNF layer are shown in Figure S3.

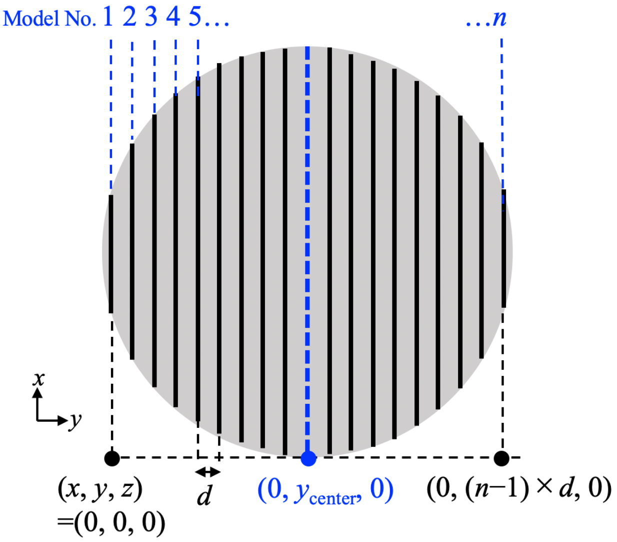

Figure S3. Coordinates of the nematic layer with multiple twisted CNF models. The straight lines represent the sweep axis (line of the center of gravity) of each model.

The y coordinate (y_{center}) of the centerline in the x-axis direction was calculated by

$$y_{\text{center}} = \frac{(n-1) \times d}{2}. \tag{S1}$$

When n is odd, the CNF model is placed on the line with $y = y_{\text{center}}$ as the sweep axis. When n is even, y_{center} is between the CNF models. The y coordinate of CNF model 1 is set to 0, so $y_{\text{center}} \neq r$.

Next, the parameters required to design the nematic layer are the coordinates of the center of gravity of the CNF model end face, length of the CNF model in the x-axis direction, and angle of rotation around the x axis in each CNF model. Each parameter is generalized

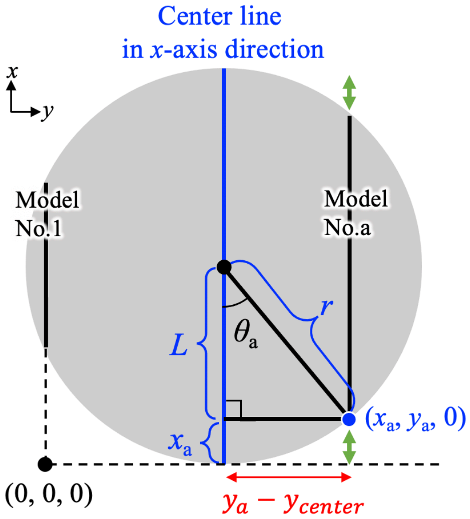

Figure S4. Illustration of calculation of each parameter of the *a*-th CNF model (model *a*).

and calculated so that the total number of CNF models n can be even or odd.

First, each parameter of the a-th CNF model is derived, as shown in Figure S4. For the coordinates of the center of gravity of the CNF model end faces

$$y_a = (a-1) \times d. \tag{S2}$$

Therefore, the distance between the centerline ($y = y_{\mathrm{center}}$) and y_a is as follows:

$$|y_a - y_{\mathrm{center}}| = |(a-1) \times d - y_{\mathrm{center}}| = |a - 1 - \frac{n-1}{2}| \times d. \tag{S3}$$

From this, the angle θ_a is

$$\sin\theta_a = \frac{y_a - y_{\mathrm{center}}}{r}, \tag{S4}$$

$$\theta_a = \sin^{-1}\left[|a - 1 - \frac{n-1}{2}| \times \frac{d}{r}\right]. \tag{S5}$$

The length of CNF model a is

$$2L = 2r\cos\theta_a. \tag{S6}$$

The x coordinate of the end-face center of gravity of CNF model a (x_a) is

$$x_a = r - L = r(1 - \cos\theta_a). \tag{S7}$$

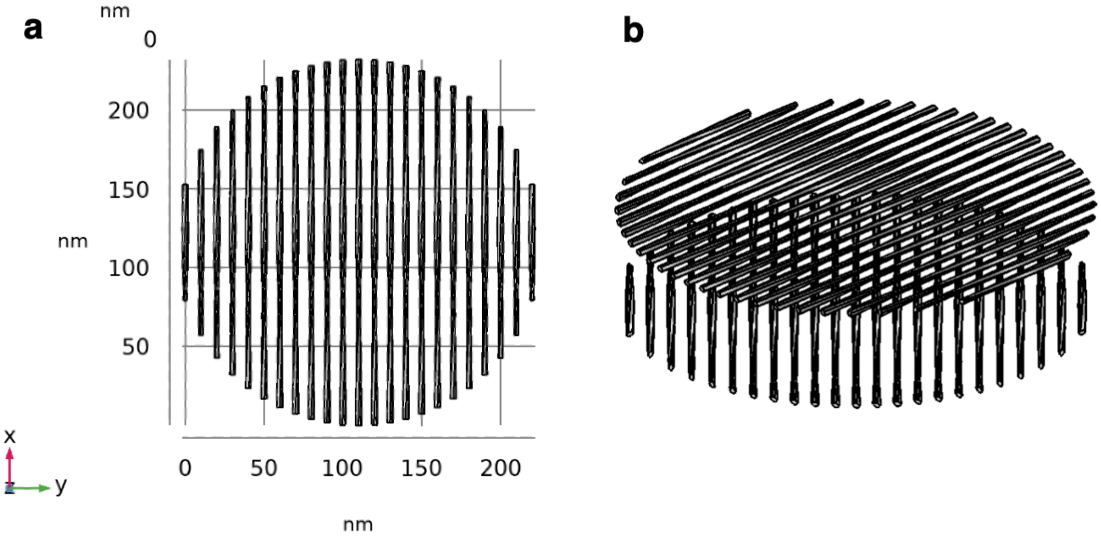

Figure S5. Demonstration structure for validation of the coordinate calculations. (a) COMSOL drawing structure of the first CNF layer when $d = 10$ nm and the number of CNF models $n = 23$. (b) COMSOL drawing structure of the structure in (a) as the first CNF layer with the second CNF layer at a distance of 50 nm in the z direction and a rotation angle around the z axis (Z_{rotate}) of −45°.

When CNF models are placed in parallel within the first CNF layer (the same relative arrangement as in Figure 4a with $X_{\text{rotate}} = 0°$), all of the CNF models must show the same cross-sectional shape in the same direction in a given yz plane. The angle of rotation for the part cut from each CNF model to be a circular array corresponds to x_a. Thus, considering a set torsion period of 2π at $2r$, the angle of rotation around the x axis that is subtracted from the CNF model per side (i.e., the angle of rotation subtracted by one length of the green double arrows in Figure S4) is

$$\pi \times \frac{x_a}{r} = \pi(1 - \cos\theta_a). \quad \text{(S8)}$$

Therefore, the angle of rotation around the x axis of CNF model a is

$$2\pi \times \frac{L}{r} = 2\pi\cos\theta_a. \quad \text{(S9)}$$

When the layers were designed, as shown in Figure S5a, for example, with $d = 10$ nm and 23 CNF models, it was confirmed that the structure was correctly constructed as designed. In particular, the cross sections of the CNF models in the same yz plane confirmed that

all of the CNF models were oriented in the same direction. The second CNF layer was a duplicate of the first CNF layer, and it was placed by setting the parallel movement distances in *x*, *y*, and *z* directions and rotation angles around the *x*, *y*, and *z* axes of the entire group. When the second CNF layer was set to +50 nm in the *z* direction and Z_{rotate} was set to −45°, we confirmed that the correct CNF model was constructed, as shown in Figure S5b.
